# Planning target volume as a predictor of disease progression in inoperable stage III non-small cell lung cancer patients treated with chemoradiotherapy and concurrent and/or sequential immune checkpoint inhibition

**DOI:** 10.1007/s10637-021-01143-0

**Published:** 2021-08-05

**Authors:** Julian Taugner, Lukas Käsmann, Monika Karin, Chukwuka Eze, Benedikt Flörsch, Julian Guggenberger, Minglun Li, Amanda Tufman, Niels Reinmuth, Thomas Duell, Claus Belka, Farkhad Manapov

**Affiliations:** 1 Department of Radiation Oncology, University Hospital, LMU Munich, Munich, Germany; 2 Member of the German Center for Lung Research (DZL), Comprehensive Pneumology Center Munich (CPC-M), Munich, Germany; 3 German Cancer Consortium (DKTK), Partner Site Munich, Munich, Germany; 4 Division of Respiratory Medicine and Thoracic Oncology, Department of Internal Medicine V, Thoracic Oncology Centre Munich, LMU Munich, Munich, Germany; 5 Asklepios Kliniken GmbH, Asklepios Fachkliniken Muenchen, Gauting, Germany

**Keywords:** Chemoradiotherapy, Checkpoint inhibition, Non-small cell lung cancer, Tumor volume, Prediction

## Abstract

*Background.* The present study evaluates outcome after chemoradiotherapy (CRT) with concurrent and/or sequential Programmed Cell Death 1 (PD-1) or Ligand 1 (PD-L1) immune checkpoint inhibition (CPI) for inoperable stage III NSCLC patients depending on planning target volume (PTV). *Method and patients.* Prospective data of thirty-three consecutive patients with inoperable stage III NSCLC treated with CRT and sequential durvalumab (67%, 22 patients) or concurrent and sequential nivolumab (33%, 11 patients) were analyzed. Different PTV cut offs and PTV as a continuous variable were evaluated for their association with progression-free (PFS), local–regional progression-free (LRPFS), extracranial distant metastasis-free (eMFS) and brain-metastasis free-survival (BMFS). *Results.* All patients were treated with conventionally fractionated thoracic radiotherapy (TRT); 93% to a total dose of at least 60 Gy, 97% of patients received two cycles of concurrent platinum-based chemotherapy. Median follow-up for the entire cohort was 19.9 (range: 6.0–42.4) months; median overall survival (OS), LRFS, BMFS and eMFS were not reached. Median PFS was 22.8 (95% CI: 10.7–34.8) months. Patients with PTV ≥ 900ccm had a significantly shorter PFS (6.9 vs 22.8 months, p = 0.020) and eMFS (8.1 months vs. not reached, p = 0.003). Furthermore, patients with PTV ≥ 900ccm and stage IIIC disease (UICC-TNM Classification 8th Edition) achieved a very poor outcome with a median PFS and eMFS of 3.6 vs 22.8 months (p < 0.001) and 3.6 months vs. not reached (p = 0.001), respectively. PTV as a continuous variable also had a significant impact on eMFS (p = 0.048). However, no significant association of different PTV cut-offs or PTV as a continuous variable with LRPFS and BMFS could be shown. The multivariate analysis that was performed for PTV ≥ 900ccm and age (≥ 65 years), gender (male), histology (non-ACC) as well as T- and N-stage (T4, N3) as covariates also revealed PTV ≥ 900ccm as the only factor that had a significant correlation with PFS (HR: 5.383 (95% CI:1.263–22.942, p = 0.023)). *Conclusion.* In this prospective analysis of inoperable stage III NSCLC patients treated with definitive CRT combined with concurrent and/or sequential CPI, significantly shorter PFS and eMFS were observed in patients with initial PTV ≥ 900ccm.

## Introduction

Lung cancer is the most frequent cause of cancer-related mortality worldwide [1]. Inoperable, locally advanced lung cancer is a very heterogeneous disease in terms of macroscopic tumor extent and patient prognosis. Historically only ten to thirty percent of these patients survive five years after multimodal treatment [2–4].

Regarding inoperable stage III NSCLC, the implementation of CPI as one of the key components of a multimodal approach has already led to an unprecedented improvement in progression-free survival (PFS) and OS [5–8]. In particular, the ground-breaking PACIFIC phase III trial demonstrated a three-year survival rate of 57% and a median PFS of 16.8 months [9, 10]. In addition, the first clinical reports on chemoradioimmunotherapy have confirmed the PACIFIC findings concerning patient outcome [7, 11–13].

Prior to the actual use of durvalumab maintenance therapy after chemoradiotherapy (CRT), planning target volume (PTV) has been considered an important prognosticator for patient outcome and treatment-related toxicity in inoperable stage III NSCLC [14–16]. Two retrospective mono-institutional analyses, in particular, reported that a PTV cut-off of 700ccm had a significant negative impact on patient outcome after conventional CRT [17, 18].

In the present prospective study, we evaluated the impact of PTV on PFS, local–regional progression-free (LRPFS), extracranial distant metastasis-free (eMFS) as well as brain-metastasis free-survival (BMFS) after CRT with concurrent and/or sequential Programmed Cell Death 1 (PD-1) or Ligand 1 (PD-L1) immune checkpoint inhibition (CPI).

## Methods

This study included data of 33 prospectively enrolled patients who received concurrent and/or sequential conventionally fractionated CRT and CPI treatment as part of a multimodal approach for inoperable UICC 8th edition stage IIIA-C NSCLC between 2017 and 2020. More precisely, CPI consisted of either sequential administration of durvalumab or conventional and sequential administration of nivolumab. All patients gave informed consent to the treatment and the prospective collection of their data for research purposes. The local ethics committee agreed to the analysis and publication of the patients’ data (17–230). All patients enrolled, were treated at a single tertiary cancer center, with either the PD-1 inhibitor nivolumab in the ETOP 6–14 NICOLAS phase II study (33%, 11 patients) or the PD-L1 inhibitor durvalumab according to the PACIFIC trial (67%, 22 patients) as part of a maintenance therapy and are henceforth referred to as the NICOLAS and PACIFIC subgroup.

Prior to treatment, radiographic imaging was performed using positron emission tomography (PET)-CT in 32 (97%) patients and CT in 1 (3%) patient. Cranial contrast-enhanced MRI was performed in 31 (94%) patients before starting treatment, two patients (6%) received contrast-enhanced cranial CT. All patients underwent pulmonary function testing and received routine blood work in order to assess kidney and liver function as well as a complete blood count. In all cases, multimodal treatment was reviewed in the multidisciplinary tumor boards. The therapeutic approach was discussed with each individual patient. Patients with an initial performance status ECOG $\ge$ 2 or poor lung function (DLCO < 40%, FEV1 < 1 L or on long-term oxygen supply) were excluded.

Based on conventional planning-CT as well as PET-CT scans in the treatment position, conventionally fractionated thoracic radiotherapy (TRT) was planned and delivered while patients were supine with their arms positioned overhead in a dedicated positioning and immobilization device - WingSTEP™ (Innovative Technologie Völp, Innsbruck, Austria). The target volumes were defined according to the European Society for Therapeutic Radiology and Oncology-Advisory Committee on Radiation Oncology Practice (ESTRO-ACROP) guidelines published in 2018 [19]. If patients were pre-treated with induction chemotherapy, only the residual primary tumor volume was contoured as gross tumor volume (GTV) and lymph node stations involved before chemotherapy were included in the clinical target volume (CTV). PTVs were generated by adding axial/cranio-caudal margins of 6/9 mm to the CTVs.

TRT was administered to the primary tumor and involved lymph nodes up to a median total dose of 63.6 Gy in 2.12 Gy single dose fractions. Radiation was delivered on a Linear accelerator (LINAC) with megavoltage capability using Volumetric Modulated Arc Therapy (VMAT) in all patients. Image-guidance was performed with a cone-beam CT at least twice a week.

All patients received a platinum-based doublet; 32 patients (97%) were treated concurrently with TRT and one patient (3%) was treated sequentially. Seventeen patients (51.5%) received at least one cycle of induction chemotherapy prior to TRT.

Durvalumab maintenance treatment at a dose of 10 mg/kg every two weeks for up to 12 months, until disease progression or the evidence of unacceptable toxicity was administered in 22 (67%) patients according to the PACIFIC trial [5, 9].

Eleven (33%) patients were enrolled in the phase II NICOLAS-trial (ETOP 6–14) and treated with concurrent nivolumab, chemotherapy and TRT, followed by nivolumab maintenance treatment every four weeks up to one year, until disease progression or the onset of unacceptable toxicity [6].

In the first two years after therapy, routine blood work, lung function testing, clinical examinations and CT or PET-CT scans were arranged every 3 months, thereafter twice a year. If clinically indicated, cranial contrast-enhanced MRI and bone-scintigraphy were additionally performed. Response was assessed according to RECIST 1.1. Local and local–regional progression (LP) along with new extracranial distant metastases (eDM) and brain metastases (BM) were documented with CT, PET-CT or MRI scans. Cytological or histological confirmation of progressive disease was not obligatory. All volumetric parameters were extracted from the radiation treatment plans. Median follow-up was calculated as the median time to loss or end of follow-up after the last day of radiotherapy in patients who were not documented as deceased. Progression-free survival (PFS) was defined as the time from the end of TRT until the occurrence of either disease progression or death. Overall survival (OS) was also calculated from the end of TRT. Time to death or metastasis (TTDM) included extracranial distant metastases (eDM) and brain metastases (BM) from the end of TRT. Univariate analysis of OS, PFS, LRPFS, eMFS and BMFS was carried out with following parameters: age, gender, T- and N-stage, histology, PD-L1 status and different PTVs. All statistics were performed using IBM SPSS version 25 (IBM, Armonk, New York, USA).

## Results

The entire cohort consisted of 33 consecutive patients with inoperable UICC 8^th^ edition stage IIIA-C NSCLC. A summary of patients’ characteristics is shown in Table 1.

**Table 1:** Patient and treatment characteristics

	N (%)
Total	33 (100)
Age median years > 65 years	62.0 15 (45.5)
Gender Male Female	24 (72.6) 9 (27.4)
T-stage 1 2 3 4	3 (9.1) 7 (21.2) 8 (24.2) 15 (45.5)
N-stage 0 1 2 3	6 (18.2) 1 (3.0) 12 (36.4) 14 (42.4)
UICC 8^th^ edition IIIA IIIB IIIC	9 (27.3) 16 (48.5) 8 (24.2)
PTV size Median ccm ≥ 900ccm	675.6 6 (18.2)
Histology -Squamous cell carcinoma (SCC) -Adenocarcinoma (AC) -Not otherwise specified (NOS)	13 (39.4) 18 (54.5) 2 (6.1)
PD-L1-status tested in ≥ 1% ≥ 50%	28 (84.8) 26 (78.8) 13 (39.4)
Radiographic imaging PET-CT cMRI	32 (97.0) 31 (93.9)
Treatment Concurrent chemoradiation (CRT) Induction chemotherapy NICOLAS PACIFIC	32 (97.0) 17 (51.5) 11 (33.3) 22 (66.7)
Median Follow-up Months after CRT	19.9
OS 6-month 12-month 18-month	100% 93.3% 87.0%
PFS 6-month 12-month 18-month	87.5% 67.7% 40.0%
eMFS 6-month 12-month 18-month	87.5% 74.2% 56%

The median age was 62.0 (range 43.8–76.9) years with 15 patients (45.5%) older than 65 years. Nine patients (27%) were female and 24 (73%) were male. In the histological evaluation, 13 (39%) patients had squamous-cell-carcinoma (SCC), 18 (55%) had adenocarcinoma (AC) and in 2 (6%) patients the tumor was classified as not otherwise specified (NOS). PD-L1 status was assessed in 28 (85%) patients prior to multimodal treatment. 26 (93% of patients tested) were listed as PD-L1 > 1% (median 60%). All 33 patients completed conventional fractionated radiotherapy to a total dose ≥ 60.0 Gy (median total dose: 63.6 Gy). Median PTV was 675.6 (range: 204.5–1234.5) ccm. Concurrent CRT was performed in 32 (97%) patients and one (3%) patient received sequential chemotherapy and TRT. The predominant concurrent chemotherapy regimen administered in 27 (82%) patients consisted of cisplatin and vinorelbine. Eleven (33%) patients were treated within the NICOLAS trial and received concomitant nivolumab (4 × 360 mg Q3W) during CRT and thereafter (480 mg Q4W) for up to one year (median cycles: 9, range: 3–14). The other 22 (67%) patients received durvalumab maintenance therapy for up to 24 cycles after the end of CRT based on the PACIFIC trial (10 mg/m^2^ Q2W; median cycles: 14, range: 2–24).

The median follow-up for the entire cohort was 19.9 (range: 6.0–42.4) months, median PFS was 22.8 (95% CI: 10.7–34.8) and median TTDM was 26.3 (95% CI: 3.6–49.0) months whereas median OS, median BMFS as well as eMFS were not reached.

For PFS, no significant difference could be revealed between the NICOLAS and the PACIFIC subgroup, with a median PFS of 22.7 (95%CI:9.1–36.4) months vs not reached (p = 0.831). The same was true for patients treated with or without induction chemotherapy, with a median PFS of 21.9 months vs not reached (p = 0.853).

Furthermore, disease stage (UICC 8th edition) had no significant impact on PFS (IIIA: median not reached, IIIB: median 22.8 months, IIIC: median 11.8 months, p = 0.810). Patients with stage IIIC disease had only a numerical inferior PFS (11.8 vs 22.8 months, p = 0.545) compared to the rest of the cohort.

No influence of PD-L1 status on PFS could be shown, neither for 0% vs. ≥ 1%

(p = 0.764, 26.3 vs 14.0 months median) nor for < 50 vs ≥ 50% (p = 0.459, 11.0 vs 14.6 months median). No significant impact of patient- (age and gender) and tumor-related (histology, T- and N-stage) characteristics on PFS was documented. For detailed results see Table 2.

**Table 2:** Results of the univariate analysis (Log-Rank test)

	Entire cohort N (%)	PFS (*p*)	LRPFS (*p*)	eMFS (*p*)
Total	33 (100)			
Age > 65 years	15 (45.5)	0.126	0.423	0.869
Gender -Male	24 (72.6)	0.484	0.746	0.901
T-stage 4	15 (45.5)	0.702	0.885	0.858
N-stage 3	14 (42.4)	0.965	0.858	0.194
UICC-stage IIIC	8 (24.2)	0.150	0.343	0.065
PTV-size ≥ 900ccm	6 (18.2)	0.020	0.064	0.003
Histology -SCC + NOS	15 (45.5)	0.708	0.413	0.842
PD-L1-status ≥ 1% ≥ 50%	26 (78.8) 13 (39.4)	0.764 0.459	0.759 0.850	0.880 0.666
Treatment Induction chemotherapy NICOLAS vs. PACIFIC	31 (93.9) 11 (33.3) vs. 22 (66.7)	0.853 0.831	0.530 0.872	0.504 0.732
Subgroup with UICC stage IIIc and a PTV ≥ 900ccm	3 (9.1%)	p < 0.001	0.123	0.001

**Fig. 1:**
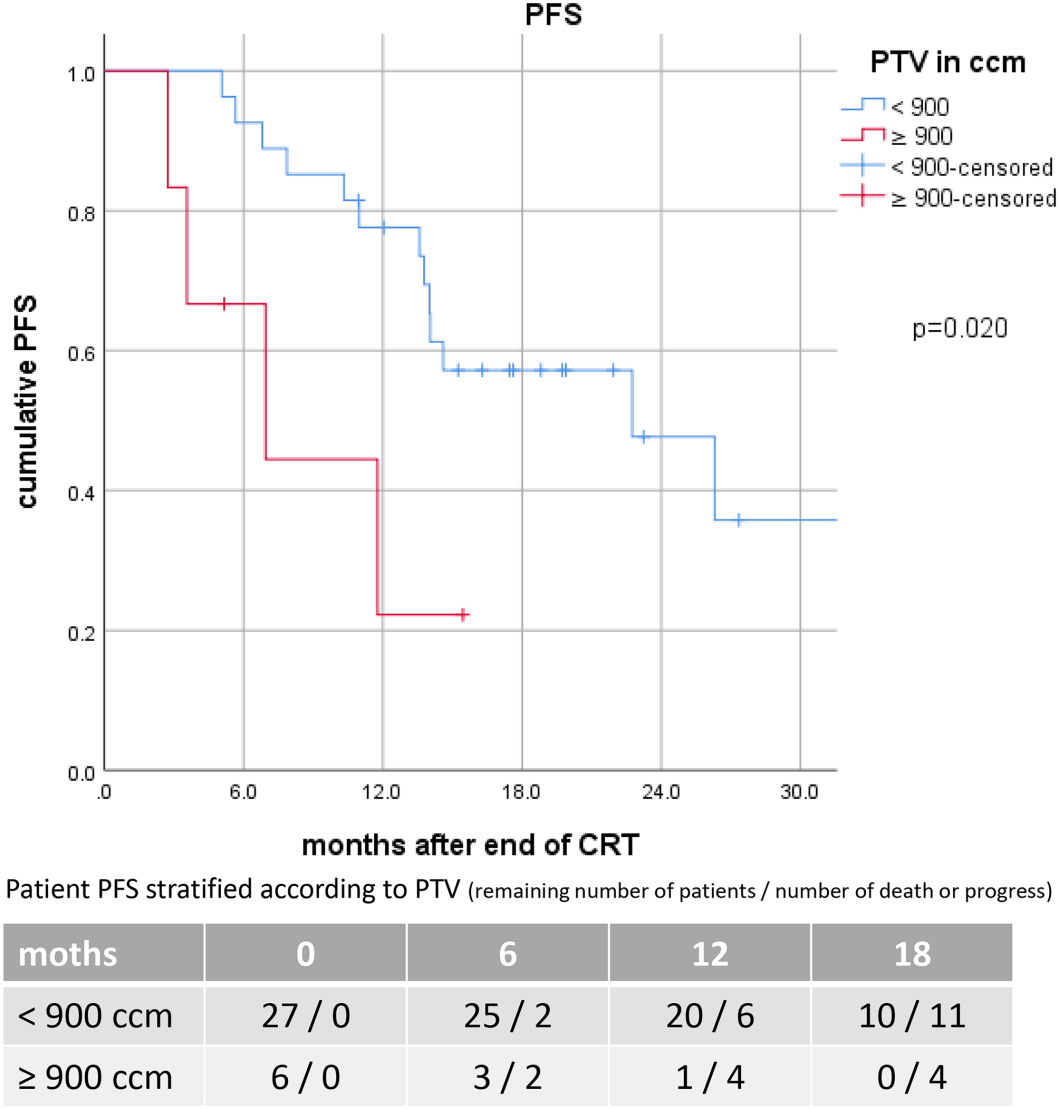
F Kaplan–Meier curves of progression-free survival (PFS) for all patients stratified according to the planning target volume (PTV)

**Fig. 2:**
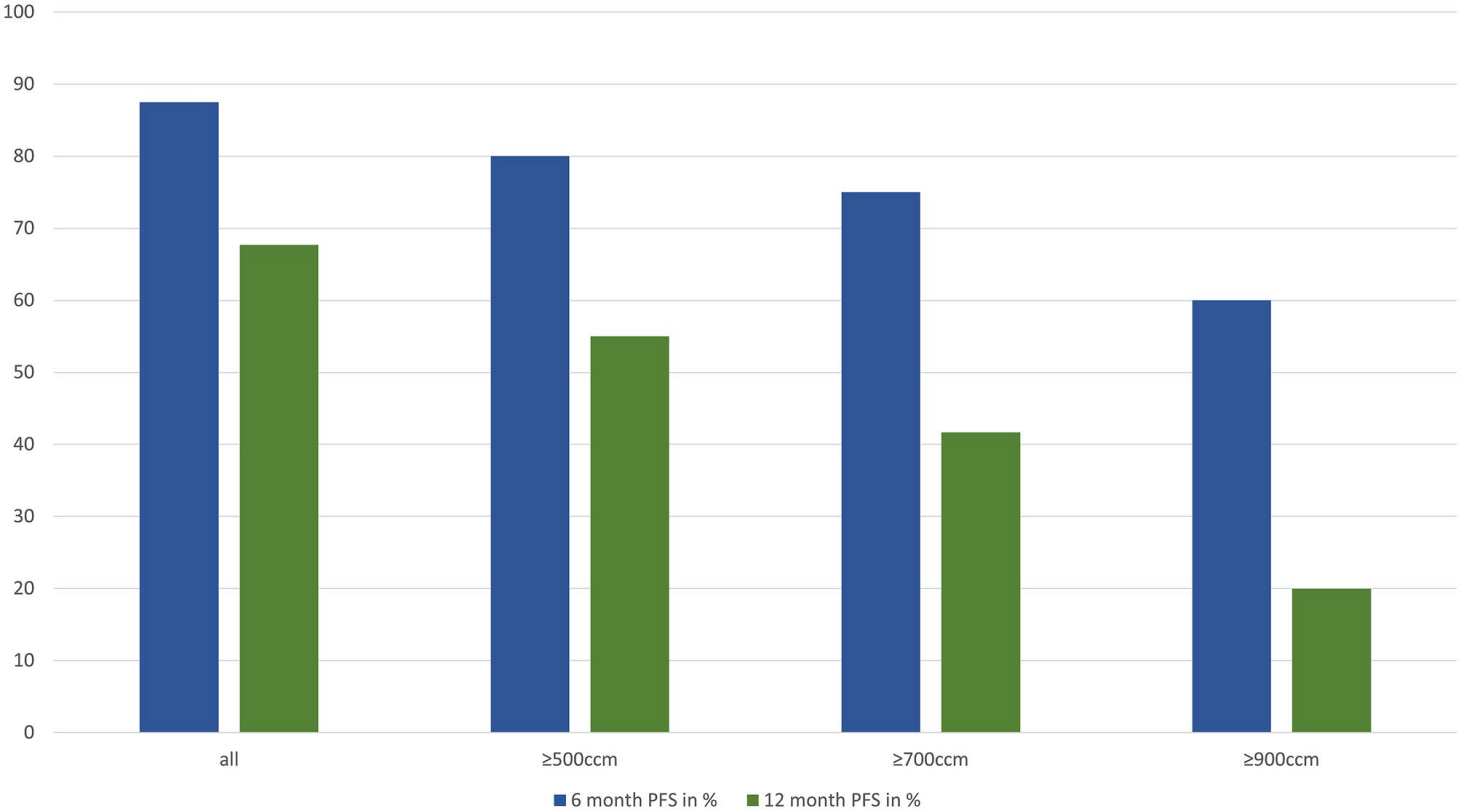
Progression-free survival (PFS) rate at 6 and 12 months regarding planning target volume (PTV)

**Fig. 3:**
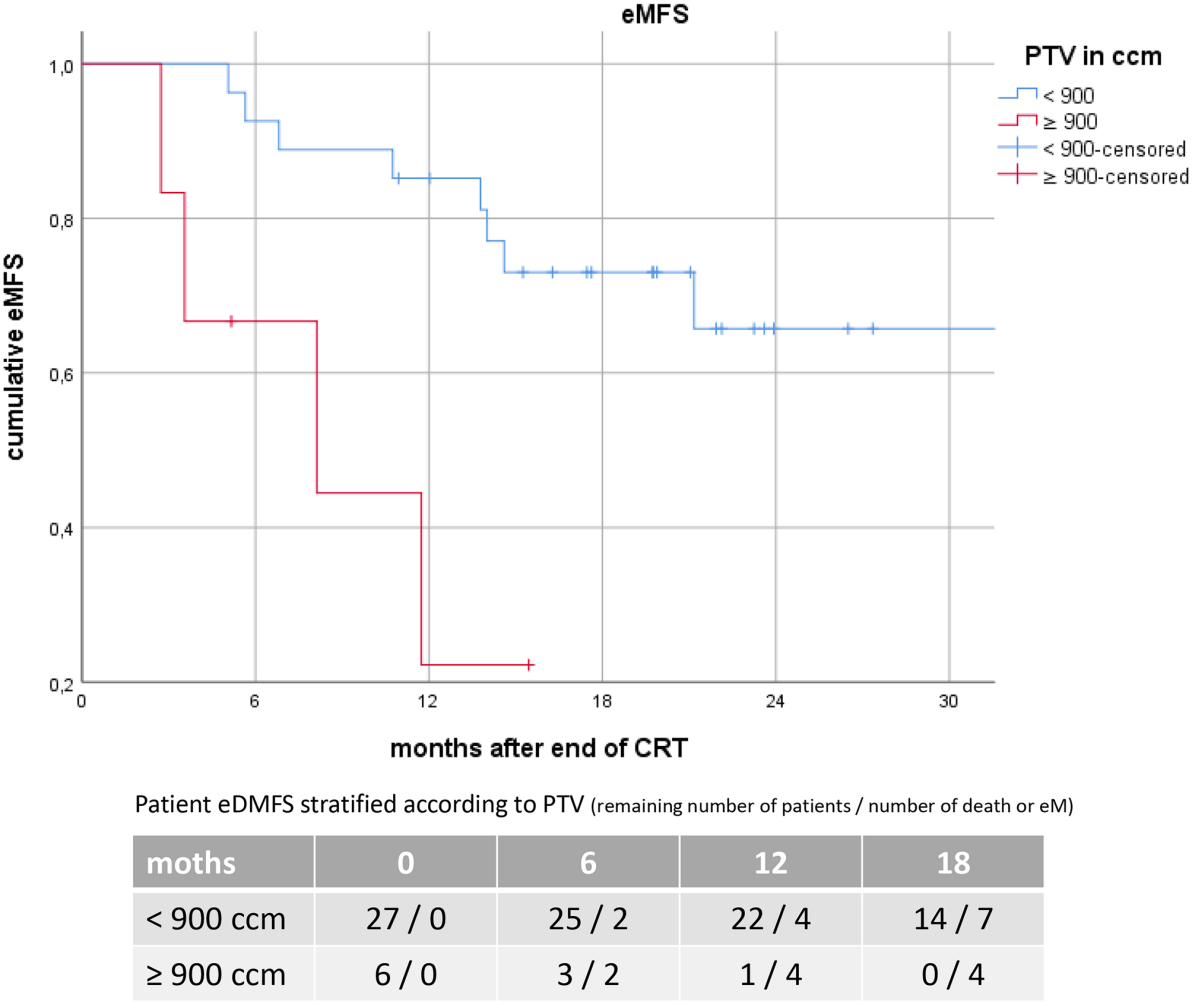
Kaplan–Meier curves of extracranial distant metastasis-free (eMFS) for all patients stratified according to the planning target volume (PTV)

However, a significant correlation between PTV and PFS was demonstrated for PTV ≥ 900ccm with a median PFS of 6.9 (95%CI: 0.3–13.6) vs 22.8 (95%CI:10.0–35.5) months (p = 0.020) Fig. 1. The corresponding 6-, 12- and 18-months PFS-rates were 60%, 20% and 0% compared to 93%, 77% and 48%, respectively Fig. 2. To further clarify the influence of PTV ≥ 900ccm as a prognostic cut-off, we also tested it for LRPFS and TTDM: A trend was observed for TTDM with a median TTDM of 8.0 vs 26.3 months (p = 0.089), but there was no significant impact on LRPFS (13.2 vs. 24.8 months, p = 0.064). We found no influence of PTV ≥ 900ccm on BMFS (15.8 vs. 32.5 months, p = 0.296). However, median eMFS was 8.1 (95%CI: 0.0–17.1) months in patients with PTV ≥ 900ccm vs not reached with PTV < 900ccm (p = 0.003) Fig. 3. The corresponding 6-, 12- and 18-months eMFS-rates were 60%, 20% and 0% compared to 93%, 85% and 66%, respectively.

Three patients (9%) with PTV ≥ 900ccm also presented with UICC stage IIIC disease; their median PFS was 3.6 (range 2.7–11.8) months after TRT in contrast to a median PFS of 22.8 (95%CI: 10.3–32.2) months in other patients (p < 0.001). Their median eMFS was 3.6 months vs not reached (p = 0.001).

In the multivariate analysis performed for PTV ≥ 900ccm as well as age (≥ 65 years), gender (male), histology (non-ACC) and T- and N-stage (T4, N3) as covariates, PTV ≥ 900ccm was the only factor that significantly correlated with PFS (HR: 5.383 (95% CI:1.263–22.942, p = 0.023)).

Moreover, we evaluated PTV as a continuous variable and discovered a significant impact on eMFS (p = 0.048; see Table 3).

**Table 3:** Outcome for PTV < 900ccm vs PTV ≥ 900ccm and results of the univariate analysis

Months after CRT	OS in % < // ≥ 900ccm	PFS in % < // ≥ 900ccm	LRPFS in % < // ≥ 900ccm	eMFS in % < // ≥ 900ccm	BMFS in % < // ≥ 900ccm
3	100//100	100//83	100//100	100//83	100//100
6	100//100	93//60	100//100	93// 60	100//100
9	100//100	85//40	96//80	89//40	96//80
12	96//80	77//20	88//60	85//20	88//60
15	92//80	56//20	75//50	72//20	79//60
	OS	PFS	LPFS	eMFS	BMFS
p-values for PTV < // ≥ 900ccm (log-rank)	0.415	0.020	0.064	0.003	0.296
p-values for PTV as a continuous variable (cox regression)	0.245	0.129	0.108	0.048	0.653

## Discussion

The aim of the present study was to evaluate the role of PTV (including the primary tumor and involved lymph node stations) on disease progression in patents with inoperable stage III NSCLC treated with CRT combined with concurrent and/or sequential CPI. Prospectively collected data of thirty-three patients were analyzed.

In accordance with the current ESTRO-ACROP guidelines for inoperable stage III NSCLC, involved lymph node stations were included in the clinical target volume (CTV). In addition, corresponding safety margins for potential patient positioning and setup errors were added in order to generate a PTV [19]. For the majority of patients, a recent PET-CT was available to delineate the target volume and if induction chemotherapy was administered in advance, imaging before and after induction was carefully considered [20].

Although these results are preliminary due to the limited number of patients and the short follow-up (median 19.9 months), we found a significantly shorter PFS and eMFS in patients with very large PTV ≥ 900ccm. For eMFS, a predictive role of PTV as a continuous variable was also revealed. Moreover, the deterioration of PFS and eMFS was more pronounced when PTV ≥ 900ccm was combined with stage IIIC disease (UICC 8th edition): In this subgroup PFS was only 3.6 (range: 2.7–11.8) months vs 22.8 (95%CI: 10.3–32.2) months in the rest of the treated cohort and eMFS was 3.6 months vs not reached (p = 0.001). Interestingly, a larger PTV was not associated with a significant increase in locoregional recurrences as well as intracranial relapse.

Historically, PTV has been a strong prognosticator regarding patient outcome in inoperable lung cancer. The Radiation Therapy Oncology Group 93–11 Phase I-II dose-escalation study confirmed an inferior PFS and OS for patients with larger tumors. In fact, patients with smaller (≤ 45cm3) tumors had a longer median survival time (MST) and a better PFS than patients with larger (> 45cm3) tumors (29.7 vs 13.3 months, p < 0.0001 and 15.8 vs 8.3 months, p < 0.0001) [21].

PTV was also validated as an important prognostic factor in the dose-escalation phase III RTOG 0617 study for inoperable stage III NSCLC [15, 16]. The open-label randomized, two-by-two factorial phase III study included 166 patients with unresectable NSCLC stage III treated with CRT between 2007 and 2011. On univariate analysis Bradley et al. indicated that increasing values of GTV and PTV are associated with increased risk of death. On multivariate analysis, PTV was among the factors predicting OS [15]. Long-term results of the RTOG 0617 trial have confirmed smaller PTV as a prognostic factor for better OS in inoperable stage III NSCLC treated with concurrent CRT [16].

Retrospective mono-institutional analyses of conventional CRT in inoperable stage III NSCLC revealed a PTV cut-off of 700ccm to have a significant impact on patient survival [17, 18].

In the PACIFIC trial, patients with inoperable stage IIIB disease (UICC 7th edition) were equally distributed between the durvalumab (44.5%) and placebo (45.1%) arm, however this trial did not provide any information about the impact of PTV on patient outcome [5, 9, 10, 22]. Shaverdian et al. [23, 24] reported no impact of PTV on patient eligibility for durvalumab maintenance therapy after CRT and no effect of PTV on the onset of pneumonitis during a durvalumab maintenance therapy.

Two studies on CRT combined with concurrent and/or sequential anti-PD-1 inhibitors (LUN 14–179 and NICOLAS) reported a significantly lower PFS and OS in patients with UICC 7th edition stage IIIB disease [6, 7, 25, 26]. In our study, the subgroup of patients with both PTV ≥ 900ccm and stage IIIC disease (UICC 8th edition) had a very short PFS and eMFS despite successfully completed trimodal therapy.

Considering the apparent limitations of our analysis, namely its single-center design, limited patient number and a median follow-up of 19.9 months, it is of potential interest to re-evaluate these findings in a larger patient collective to confirm an impact of PTV on the course of disease and long-term patient outcome.

In summary, the present results show a significant deterioration of PFS and eMFS in inoperable stage III NSCLC patients with very large PTV (≥ 900ccm). This negative effect was more pronounced in patients with stage IIIC disease (UICC 8th edition). Our findings suggest a potential role for induction treatment in this subgroup of patients. Several studies investigating induction therapy for definitive CRT combined with concurrent and/or sequential CPI are ongoing or planned [27–31].

## Conclusion

The present study revealed that PTV ≥ 900 cc has a significant impact on PFS and eDMFS in inoperable stage III NSCLC patients treated with definitive CRT combined with concurrent and/or sequential CPI.

## Data Availability

The data that support the findings of this study are available from the corresponding author, upon reasonable request.
